# Supplementary material for: The role of catalytic and regulatory domains of human PrimPol in DNA binding and synthesis
Source: Nucleic Acids Res. 2023 Jun 16;51(14):7541–51. doi: 10.1093/nar/gkad507 (PMC10415149; doi:10.1093/nar/gkad507)
Supplement: gkad507_Supplemental_File [file gkad507_supplemental_file.pdf]

Supplementary information

The role of catalytic and regulatory domains of human PrimPol in DNA binding and synthesis

Elizaveta O. Boldinova[1,2#], Andrey G. Baranovskiy[3#], Diana I. Gagarinskaya[1,3], Anna A. Manukyan[1,2], Alena V. Makarova[1,2]*, Tahir H. Tahirov[3]*

[1] Institute of Molecular Genetics, National Research Center «Kurchatov Institute», Kurchatov sq. 2, 123182, Moscow, Russia
[2]Institute of Gene Biology, Russian Academy of Sciences, Vavilov 34/5, 119334, Moscow, Russia
[3]Eppley Institute for Research in Cancer and Allied Diseases, Fred & Pamela Buffett Cancer Center, University of Nebraska Medical Center, Omaha, Nebraska 68198, United States

# equal contribution

* corresponding author

E-mail address: amakarova-img@yandex.ru (A.V. Makarova); ttahirov@unmc.edu (T.H. Tahirov)

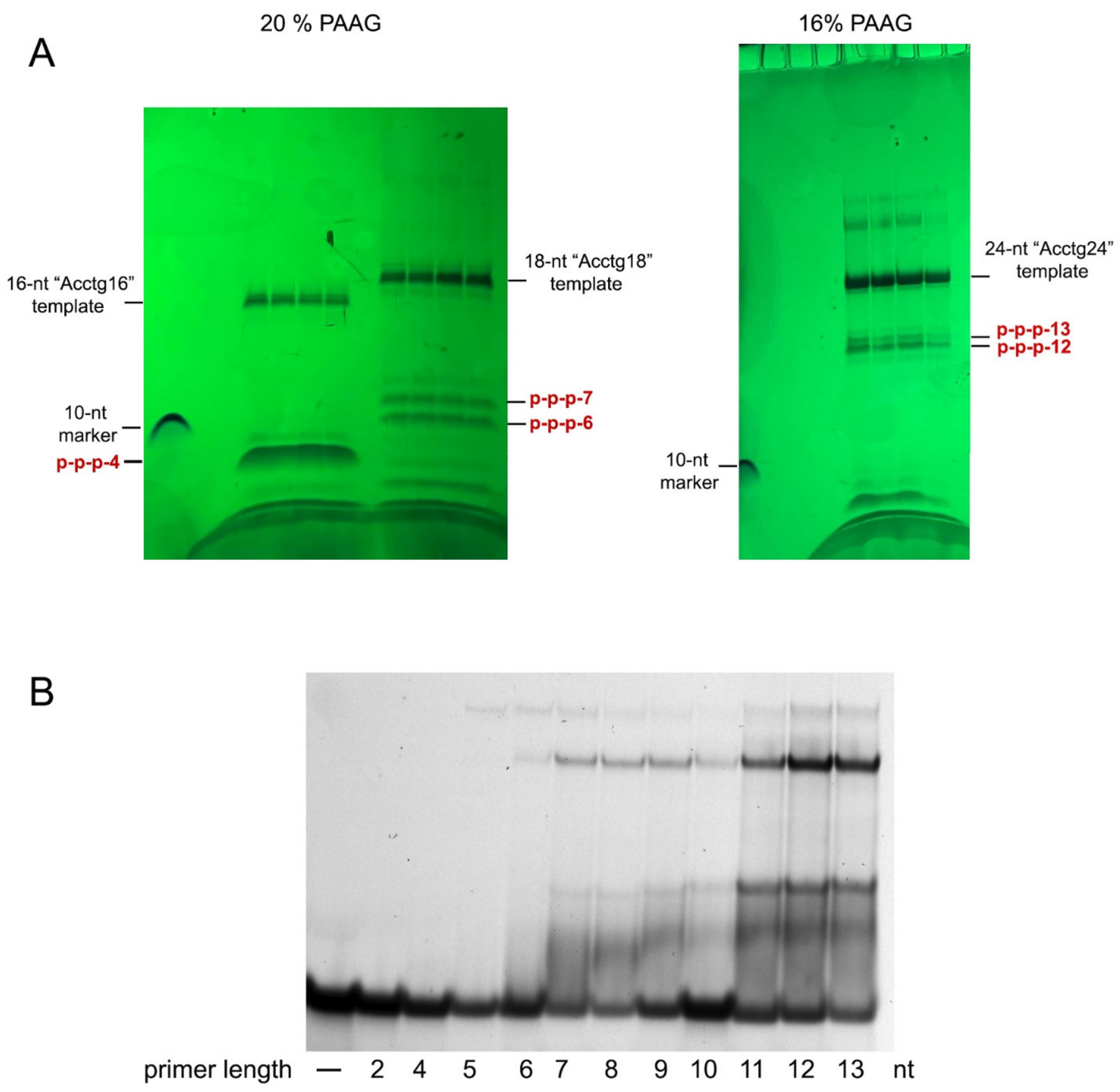

Figure S1. (**A**) Examples of isolation of oligonucleotides with the 5`-triphosphate from the 16% (4-, 6- and 7-mer) or 20% (12- and 13-mer) Urea-PAGE. DNA primase reactions with PrimPol and Acctg24 template or corresponding shorter templates (Acctg18, Acctg16) were carried out in the presence of both Mn^{2+} and Mg^{2+} ions. The products of DNA primase reactions were loaded on a gel and visualized by UV shadowing on a fluorescent TLC plate. (**B**) EMSA of PrimPol binding to a DNA template annealed to primers of different length containing the 5`-triphospate. Reactions were incubated with 2 µM PrimPol in the presence of 300 nM DNA and 1mM Mn^{2+}, pH 7.0 at 8ºC. Short DNA duplexes (containing 2-6 mers) are not stable. The reason for poor PrimPol binding to DNA with a 10-mer primer is not clear and can be due to a clash between NTD and CTD.

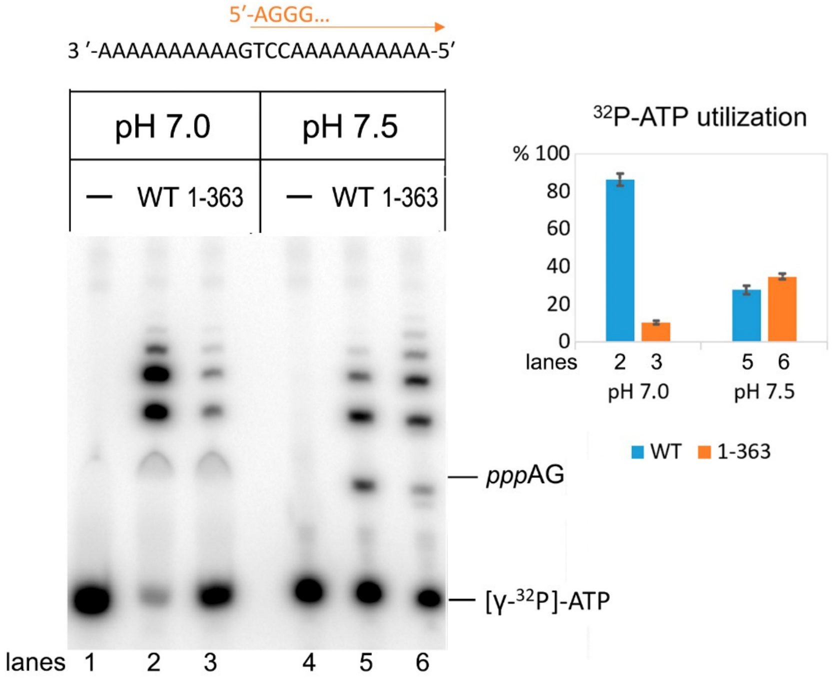

Figure S2. The DNA primase reactions with the full-length PrimPol and $PrimPol_{1-363}$ variant (NTD) were carried out in the presence of 1 mM Mn^{2+} ions, [γ-^{32}P]-ATP, rATP and dGTP at pH 7.0 or 7.5. PrimPol makes an A-G mismatch upon the third and following dGMP insertions.

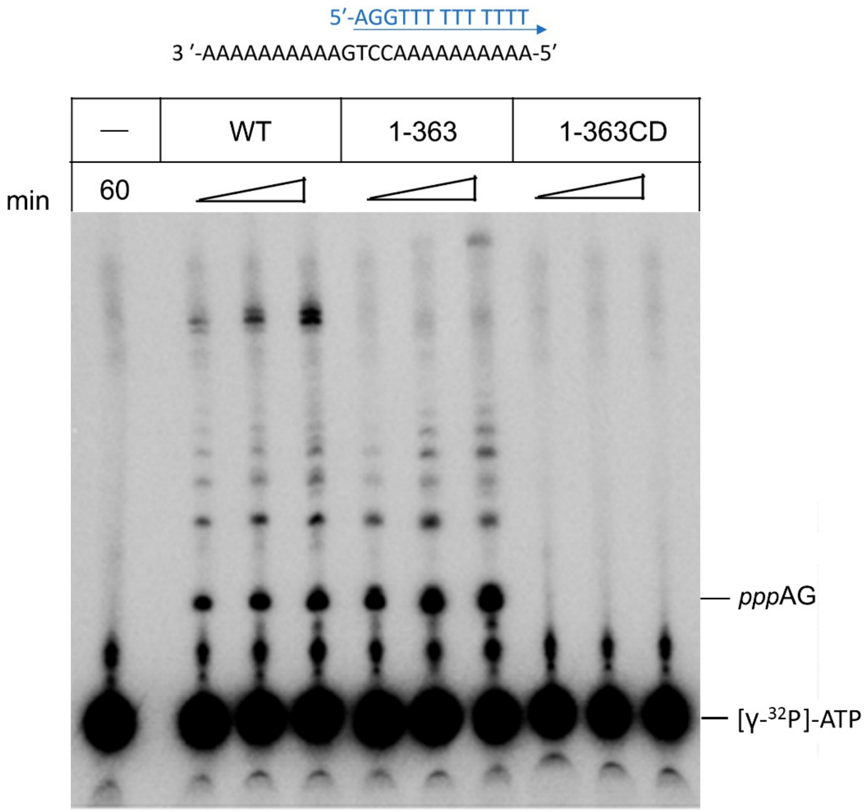

Figure S3. The DNA primase reactions with the full-length PrimPol, $PrimPol_{1-363}$, and $PrimPol_{1-363CD}$ variants were carried out at pH 7.5 in the presence of 1 mM Mn^{2+} ions, [γ-^{32}P]-ATP, rATP, dGTP and dTTP; 15 – 60 min incubation time.

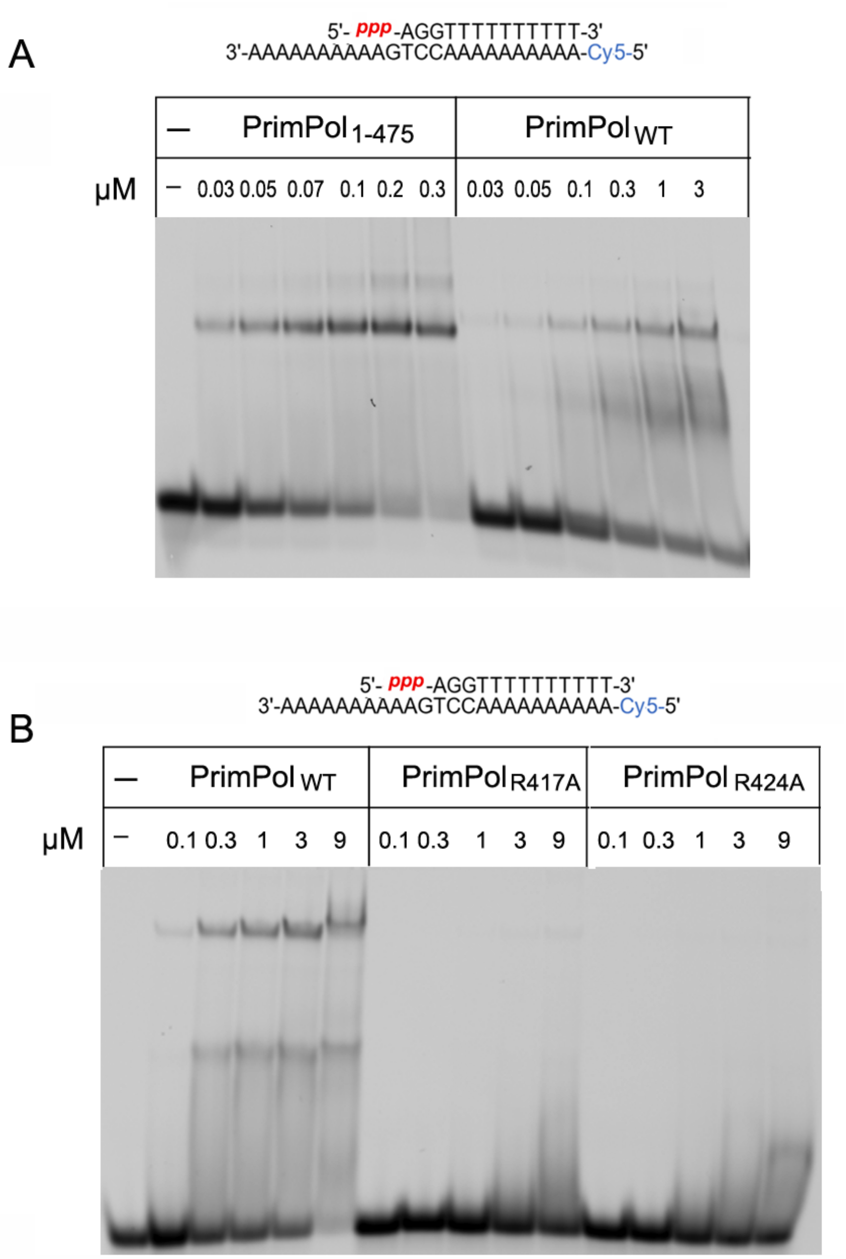

Figure S4. Affinity analysis of the $PrimPol_{WT}$, $PrimPol_{1\text{-}475}$ (**A**) and $PrimPol_{WT}$, $PrimPol_{R417A}$, $PrimPol_{R424A}$ (**B**) variants for a DNA template annealed to a 12-mer primer with a 5`-triphospate determined by EMSA. Reactions were incubated with 0.03 – 9 µM PrimPol in the presence of 300 nM DNA and 1mM Mn^{2+}, pH 7.0.

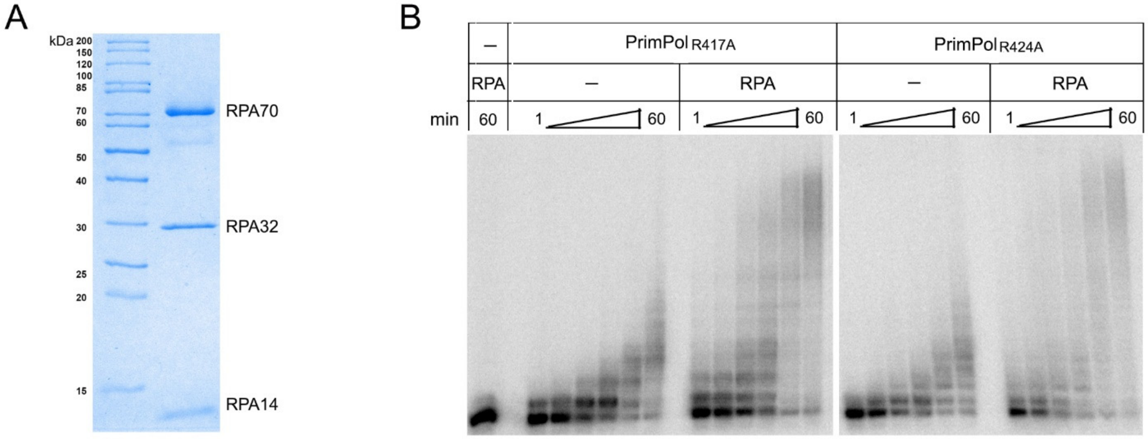

Figure S5. The regulation of PrimPol by RPA. (**A**) Purified RPA. Coomassie staining of 13% PAAG. (**B**) The stimulation of the DNA polymerase activity of $PrimPol_{R417A}$ and $PrimPol_{R424A}$ by RPA. When applicable, the ^{32}P-labeled Template55/Primer-18 substrate was preincubated with 10 nM RPA at 30ºC for 3 min. Reactions were started by adding 100 nM PrimPol and incubated for 1–60 min; pH 7.0 and 10 mM Mg^{2+}.
